# Factors Associated with Adverse Neonatal Outcomes in Complete Rupture of the Pregnant Uterus: A Single-Center Cohort Study

**DOI:** 10.3390/jpm16060327

**Published:** 2026-06-18

**Authors:** Bohye Gil, Sohyun Shim, Yoon Jang, Joong Sik Shin, Nara Lee, Mi Kyoung Kim, Yong Wook Jung, Seok Ju Seong, Mi-La Kim

**Affiliations:** Department of Obstetrics and Gynecology, CHA Gangnam Medical Center, CHA University, Seoul 06135, Republic of Korea; docgil@chamc.co.kr (B.G.); simuso@chamc.co.kr (S.S.); b263004@chamc.co.kr (Y.J.); shinjs@cha.ac.kr (J.S.S.); naradd@chamc.co.kr (N.L.); ra13811@chamc.co.kr (M.K.K.); dumbung@chamc.co.kr (Y.W.J.); sjseong@cha.ac.kr (S.J.S.)

**Keywords:** complete uterine rupture, scarred uterine rupture, unscarred uterine rupture, neonatal outcomes

## Abstract

**Objective**: This study aimed to evaluate clinical characteristics and outcomes of complete uterine rupture during pregnancy and identify factors associated with adverse neonatal outcomes. **Methods**: This retrospective cohort study analyzed data from a single center between January 2008 and July 2024. Complete uterine rupture was defined as full-thickness myometrial and serosal rupture confirmed during surgery. **Results:** Among 50,185 deliveries, 22 cases of complete uterine rupture were identified (incidence: 0.044%). Most patients (86.4%) had a scarred uterus, exclusively due to previous myomectomy (*n* = 19). While abdominal pain was the primary symptom (72.7%), 22.7% of patients were asymptomatic. There were no cases of maternal mortality or peripartum hysterectomy. Of the 25 neonates, 12 (48%) experienced adverse outcomes, defined as NICU admission or perinatal death. Adverse neonatal outcomes were significantly associated with preterm delivery (*p* = 0.030), fetal heart rate abnormalities (*p* = 0.040), and a prolonged symptom-to-delivery interval (*p* = 0.032). Univariate analysis identified preterm delivery and abdominal pain as significant predictors of poor neonatal prognosis. **Conclusions**: Complete uterine rupture is a rare but critical obstetric emergency. Although maternal outcomes were favorable in this study, nearly half of the neonates experienced adverse outcomes. Preterm labor and abdominal pain serve as significant prognostic indicators. These findings emphasize that early clinical suspicion and minimizing the time from symptom detection to delivery are vital for optimizing neonatal survival and health.

## 1. Introduction

Uterine rupture is a potentially catastrophic obstetric event with serious maternal and fetal consequences. The overall incidence of uterine rupture is 1.6–7.8 in 10,000 births [1]. In females with scarred uteri, the incidence is higher, ranging from 1 in 100 to 1 in 200 deliveries [2]. Although advances in medical accessibility and diagnostic tools have greatly reduced mortality by accelerating diagnosis and treatment, patients must be aware of dangerous situations when encountering a uterine rupture. If not swiftly managed, maternal complications, such as massive hemorrhage, multiple blood transfusions, hysterectomy, associated visceral injuries, disseminated intravascular coagulation (DIC), sepsis, and shock, can occur. In fetuses and neonates, uterine rupture can lead to fetal hypoxia/anoxia, sepsis, and neonatal mortality [3].

With increasing maternal age, cesarean delivery rates, and myomectomy rates in females, obstetricians are increasingly exposed to the risk of uterine rupture. Myomectomy scars may lead to rupture due to improper healing, residual weakness, or disruption of the myometrial fiber architecture, which behaves differently from the lower uterine segment scar of a cesarean section. Clinical manifestations of uterine rupture include abnormal fetal heart rate, acute vaginal bleeding, abdominal pain, and hemodynamic instability. However, these patients are sometimes asymptomatic [4]. While numerous studies focused on the clinical presentation of uterine rupture, risk factor analyses of adverse neonatal outcomes remain limited.

This study aimed to evaluate the clinical characteristics and obstetric and neonatal outcomes of patients with complete uterine rupture during pregnancy and to identify factors associated with adverse neonatal outcomes in complete uterine rupture based on data from a single center.

## 2. Materials and Methods

A retrospective review was performed on patients delivered at >20 weeks of gestational age and who were diagnosed with complete uterine rupture at the CHA Gangnam Medical Center between January 2008 and July 2024. Complete uterine rupture, confirmed during surgery, was defined as a full-thickness rupture of the myometrium and uterine serosa, whereas dehiscence refers to a separation of the prior scar without fetal expulsion into the peritoneal cavity. In this analysis, uterine dehiscence, defined as a partial myometrial disruption with intact serosa, was excluded [5].

The study protocol was approved by the Institutional Review Board of CHA Gangnam Medical Center (GCI-2024-09-004), which granted a waiver of informed consent. Data collected through medical chart review included age, body mass index, parity, mode of pregnancy, history of uterine surgery (including cesarean delivery, myomectomy, surgery of cornual pregnancy, or perforation during hysteroscopic surgery), gestational age at delivery, mode of delivery, twin pregnancy, associated uterine anomalies, symptoms and signs of uterine rupture (including abdominal pain, vaginal bleeding, hemodynamic instability, and fetal heart rate abnormality), site of uterine rupture, time interval of symptoms and signs to delivery or fetal demise (FD) detection. The time interval between the onset of clinical signs or symptoms and delivery was calculated to assess the promptness of emergency management. For patients admitted through the emergency department, the onset was defined as the time of admission. For patients who were already hospitalized at the time of the event, the onset was recorded based on the earliest timestamp of symptom or sign documentation in the nursing charts. The final diagnosis was confirmed by visual inspection of the uterine rupture via laparotomy. To evaluate maternal and neonatal outcomes, data on estimated blood loss, peripartum blood transfusion, organ injury, hysterectomy, DIC, maternal mortality, neonatal birth weight, Apgar scores at 1 and 5 min, neonatal intensive care unit (NICU) admission, and perinatal mortality (including FD) were collected from medical chart review. NICU admission was determined based on standardized institutional protocols for neonates exhibiting significant morbidity, including acute respiratory distress, persistent fetal heart rate abnormalities, hypoxic–ischemic encephalopathy, or the need for intensive monitoring due to clinical instability following uterine rupture. This ensures that NICU admission reflects significant neonatal morbidity rather than elective or non-emergent care. In the case of a twin pregnancy, data collected from the first baby were used for analyzing neonatal outcomes.

All statistical analyses were performed using Statistical Package for Social Sciences software version 26.0 (International Business Machine Corp., Armonk, NY, USA). Descriptive data are expressed as mean ± standard deviation and median, range. Quantitative variables were compared using the Mann–Whitney U test according to the Shapiro–Wilk test for normal distribution. Categorical variables were compared using Fisher’s exact test and chi-square test. Logistic regression was used to calculate the odds ratio (OR), presented with 95% confidence intervals (CIs), to evaluate the factors associated with adverse neonatal outcomes. Statistical significance was set at *p*-value < 0.05.

## 3. Results

**Figure 1:**
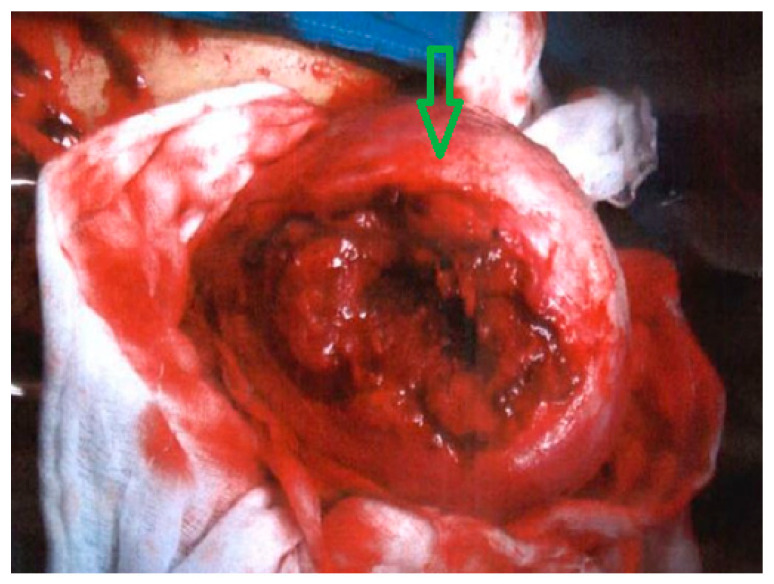

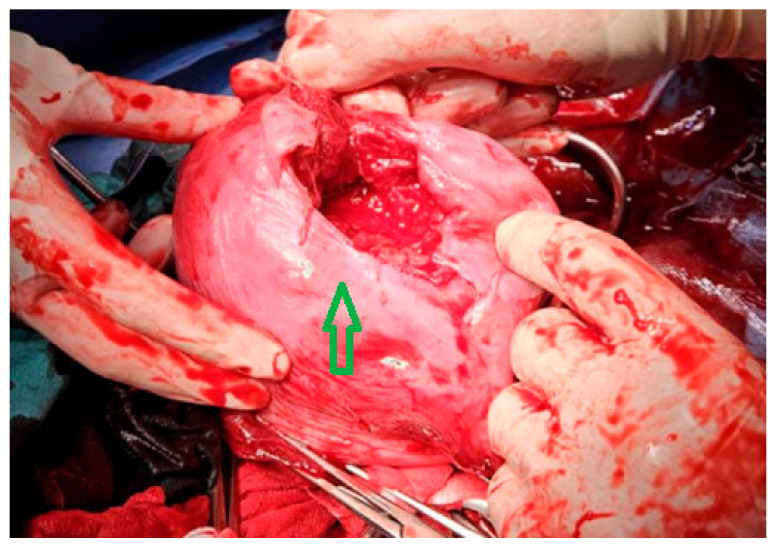
Operative findings of complete uterine rupture. The arrow indicates the site of uterine rupture.

During the study period, 22 cases of complete uterine rupture were identified among 50,185 deliveries at >20 weeks of gestation, corresponding to an incidence rate of 0.044%. Figure 1 presents a case of complete uterine rupture from this study.

The baseline characteristics of the patients with scarred and unscarred uteri are presented in Table 1. Among the 19 patients with scarred uterus, previous myomectomy was the most common type of uterine surgery (10 of 19). Three patients had an unscarred uterus, all of whom experienced a trial of labor. Two underwent cesarean delivery due to progress failure at 3 cm and full dilatation of the cervix, while one was diagnosed postpartum due to massive postpartum bleeding after vaginal delivery. Abdominal pain was the most common symptom, which occurred in 16 patients (72.7%), while 5 patients (22.7%) had no specific symptoms. Gestational age at delivery was significantly higher in the unscarred uterus group (*p* = 0.01). Cesarean delivery was more frequent in the scarred uterus group as the mode of delivery (*p* = 0.01), while the lower uterine segment was the most common site of rupture in the unscarred group (*p* = 0.02). However, the preterm delivery rate was not statistically significant between groups (*p* = 0.214).

Table 2 represents the maternal and neonatal outcomes for the scarred and unscarred uterus groups. No statistically significant differences were observed between the variables. Notably, no hysterectomy or maternal mortality occurred during the study period. Four cases of perinatal mortality, including two cases of FD, were reported, all in the scarred uteri group; however, the difference between the groups was not statistically significant (*p* = 1.000).

As shown in Table 3, neonatal outcomes were categorized into two groups: no neonatal complications and those involved in NICU admission or perinatal mortality. A total of 12 cases (54.5%) involved NICU admission or perinatal mortality, including FD. Factors significantly associated with these adverse neonatal outcomes were younger gestational age at delivery (*p* = 0.006), preterm delivery (*p* = 0.030), signs of fetal heart rate abnormality (*p* = 0.040), and a longer time interval of symptoms and signs of delivery or FD detection (*p* = 0.032). No significant differences in adverse neonatal outcomes were observed between scarred and unscarred uteri (*p* = 0.571), sites of uterine rupture (*p* = 0.726), or twin pregnancies (*p* = 0.221).

In the univariate analysis using logistic regression (Table 4), factors significantly associated with adverse neonatal outcomes were preterm delivery (OR, 12.000; 95% CI, 1.581–91.084, *p* = 0.016) and symptoms of abdominal pain (OR, 11.000; 95% CI, 1.005–120.430, *p* = 0.050). However, in logistic regression analysis, the time interval between symptoms and signs of delivery or FD detection was not significantly associated with adverse neonatal outcomes (OR, 0.986; 95% CI, 0.997–1.001, *p* = 0.072).

## 4. Discussion

Uterine rupture is a rare but potentially catastrophic obstetric complication that poses significant risks to both the mother and the fetus. The incidence of uterine rupture differs by country: reported as 0.078% in Finland, 0.016% in Austria, and 0.059% in the Netherlands [6]. In South Korea, data on uterine rupture are based on case reports, with no available reports on its incidence. In our study, 22 cases of complete uterine rupture among 50,185 deliveries at ≥20 weeks of gestation yielded an incidence rate of 0.044%.

Among the various associated factors for uterine rupture, the most commonly recognized is the presence of a uterine scar, particularly from a previous cesarean delivery [7]. However, this study identified prior myomectomy as the most common uterine scar, which differs from the patterns observed in other countries. This discrepancy can be attributed to differing practices regarding vaginal birth after cesarean section (VBAC). In many countries, VBAC is commonly attempted unless there is an absolute contraindication [8]. Conversely, in South Korea, VBAC is performed only in rare and highly selective cases, primarily due to concerns regarding potential lawsuits and a general preference for elective repeat cesarean delivery. Consequently, when VBAC is attempted, it is restricted to highly selective cases, typically involving patients with a single prior low-transverse cesarean section, spontaneous onset of labor, and no contraindications such as a history of classical or T-shaped uterine incisions, prior uterine rupture, or obstetric complications requiring immediate delivery. This clinical landscape contrasts significantly with many Western countries, where VBAC is more frequently offered. Such differences in obstetric practice contribute to the unique profile of our study population, where the history of prior myomectomy is more prevalent than a history of prior cesarean delivery as a predisposing factor for uterine rupture. Consequently, in our study, 52.6% (10 of 19) of the patients had a history of previous myomectomy, whereas only 10.5% (2 of 19) had prior cesarean delivery.

Our findings should be contextualized within the broader international obstetric landscape. According to the International Network of Obstetric Survey Systems (INOSS) study, the prevalence of complete uterine rupture is significantly influenced by regional VBAC rates [1]. Specifically, the INOSS study observed that in Western cohorts, the risk of rupture is predominantly driven by VBAC in patients with a history of previous cesarean delivery. In contrast, our cohort demonstrates a distinct clinical profile; given the extremely low prevalence of VBAC in South Korea, uterine rupture cases are more frequently associated with non-cesarean uterine histories, such as prior myomectomy. This discrepancy highlights that the risk factors for uterine rupture are deeply rooted in regional obstetric practice patterns, where medico-legal considerations and strong patient preferences often shift the focus away from VBAC-related ruptures. Consequently, clinicians should maintain a high index of suspicion for uterine rupture even in patients without a history of cesarean section, particularly those who have undergone previous uterine surgery such as myomectomy.

The largest study on infant outcomes after complete uterine rupture was conducted by Al-Zirqi et al. in 2018 [8]. This population-based study included 244 births from complete ruptures in 2,455,797 births over 41 years of duration [8]. Infant outcomes were categorized into four groups: 44.7% of the infants were born healthy without the requirement of NICU care, 23% required NICU admission, 26.2% had intrapartum or infant mortality, and 6.1% were categorized as having hypoxic–ischemic encephalopathy [8]. In our study, owing to the relatively small number of participants, we divided the data into two groups: no neonatal complications (45.5%) and NICU admission or perinatal mortality (54.5%). The infant mortality rate was 18.2%. Notably, in both studies, uterine scarring did not affect infant mortality. Additionally, the longer the time interval to delivery, the lower the possibility of having a healthy baby. According to Al-Zirqi et al., when the time-to-delivery interval was >30 min, mortality increased by 16.7 times compared to neonates born within <20 min. In addition, each additional minute results in a 5% decrease in the chances of delivering a healthy baby [8]. At our institution, the average time interval from the onset of symptoms or signs to delivery was 42 min in the group with healthy outcomes compared to 111 min in the group requiring NICU admissions or perinatal mortality. The reason for the difference in time between the two studies is, as mentioned earlier, that VBAC is rarely performed in South Korea, whereas in other countries, the most common causes of uterine rupture are previous cesarean delivery and labor induction. According to another study reported by Al-Zirqi et al., in Norway, a trial of labor after cesarean delivery was attempted in 64% of cases [9]. Labor is commonly induced at full term, and since the risk of rupture in VBAC is well known, delivery units are typically prepared for any immediate emergency cesarean delivery if necessary. However, data from our institution mostly involved patients with uterine scars from myomectomy, who were either presented with abdominal pain or were admitted for preterm labor and were found to have uterine rupture. The rate of full-term and overterm deliveries in the comparative study was 95.0%, whereas in our study, the rate of full-term deliveries was only 50.0%.

Factors significantly related to adverse neonatal outcomes, as shown in Table 4, included preterm delivery and abdominal pain. Unlike the associated factors compared in Table 3, the OR obtained from logistic regression revealed that the time interval was marginally significantly related to the adverse outcomes. Based on these findings, deciding on emergency cesarean delivery in preterm is difficult. While delivery in preterm itself may result in poor neonatal outcomes, hesitation in performing cesarean delivery when abdominal pain is present might miss the optimal window for fetal viability. The timing is especially difficult compared to other acute abdominal diseases since obstetric diagnosis mainly relies on the patient’s history, clinical presentation, cardiotocography, and ultrasonography. When blood flow around the rupture site is weak or there is adhesion coverage, the patient may be asymptomatic until the rupture is identified during surgery. Similarly, the timing of uterine rupture is unpredictable, and the rupture site may be irrelevant to the original surgical site. Abdominal pain can be misdiagnosed as other acute abdominal conditions or early labor symptoms. Furthermore, conditions that do not directly lead to uterine rupture may obscure early recognition, such as intellectual disability, history of abdominal trauma, use of ritodrine, or hyperemesis during pregnancy [10]. Tachycardia, if not addressed promptly, can lead to shock or maternal mortality. In two cases from our data, patients exhibited tachycardia and abdominal pain while being treated for preterm labor with ritodrine and were later diagnosed with uterine rupture. Clinicians should be aware that the use of ritodrine may mimic or mask the early clinical symptoms of uterine rupture, potentially delaying timely diagnosis and intervention. Sonographic findings, along with clinical correlation and serial laboratory tests, guide the decision to perform an emergency laparotomy.

Globally, there has been a gradual increase in the proportion of women conceiving at an older age, leading to an increase in myomectomies before pregnancy or assisted reproductive technology (ART) procedures [11]. Furthermore, as the number of patients undergoing ART increases, the number of patients requiring salpingectomy or cornual resection due to abnormal embryo implantation also increases. Consistently, women with a history of cornual resection accounted for 27.6% (six cases) of our findings, which is a notable proportion. These finding warrants careful consideration of the unique anatomical and physiological characteristics of this region. The cornual area is a complex transition zone where the fallopian tube joins the uterine cavity, characterized by a narrower myometrial layer compared to the uterine corpus. This anatomical site is particularly susceptible to structural compromise following surgical interventions, such as cornual resection for ectopic pregnancy, salpingectomy, or myomectomy. Furthermore, in patients who have undergone ART, the cornual region is subjected to significant hormonal stimulation and uterine expansion, which may exacerbate the vulnerability of pre-existing surgical scars or tissue thinning in this region. This confluence of anatomical narrowness, surgical trauma history, and the physiological stress of pregnancy appears to contribute to the increased risk of rupture observed in our study.

Several methodological considerations warrant careful interpretation of our findings. Primarily, the use of a composite outcome, comprising both NICU admission and perinatal mortality, was necessitated by the extreme rarity of complete uterine rupture. Notably, NICU admissions at our institution are strictly dictated by standardized protocols for clinical instability, ensuring that these metrics reflect significant medical necessity rather than elective intervention. While the clinical heterogeneity of this composite outcome is acknowledged, it remains the most appropriate measure to capture the full spectrum of severity in the context of such catastrophic events. Given the rarity of complete uterine rupture and the limited sample size (*n* = 22), this composite endpoint provides a necessary statistical approach to demonstrate the overall neonatal burden. Nevertheless, as perinatal death represents the most catastrophic manifestation of this injury, findings should be interpreted with due consideration of the distinct clinical implications between neonatal morbidity and mortality.

A strength of our study is that it reflects the current situation in South Korea, where there are few reports on associated factors for uterine rupture and neonatal outcomes, particularly given that VBAC is rarely performed. Based on these findings, warnings regarding preterm delivery and uterine rupture are important. However, this study had a few limitations. First, this retrospective design inherently limits the completeness and consistency of data collection. Additionally, not all patients included in the study underwent myomectomy or corneal resection at our institution, resulting in the limited availability of detailed surgical information. Consequently, the impact of these surgical factors could not be thoroughly analyzed. Furthermore, several statistical limitations must be acknowledged when interpreting our findings. The small sample size (*n* = 22) significantly constrained our ability to perform multivariable logistic regression. This resulted in unstable odds ratios for certain variables with sparse data. To ensure scientific rigor and avoid overinterpretation, we have explicitly marked these results as ‘Not Available’ (N/A) in Table 4. Consequently, the observed associations between clinical variables and adverse neonatal outcomes should be interpreted as exploratory rather than definitive. Future studies with larger multicenter cohorts are warranted to validate these potential predictors and to establish more robust clinical models for risk stratification in cases of complete uterine rupture. In addition, due to the small number of cases, multivariate analysis could not be performed, limiting the ability to comprehensively identify and evaluate the factors associated with adverse neonatal outcomes. Future larger-scale prospective studies that incorporate detailed surgical data are essential to address these limitations and provide more robust insights.

Beyond the technical aspects of emergency management, the clinicians’ burden on patients and their families in such catastrophic situations cannot be overlooked. In cases of uterine rupture, particularly when associated with adverse neonatal outcomes, the communication strategy and skills of all healthcare professionals involved are of paramount importance. Fostering a patient-centered approach and providing empathetic support should be integrated into the comprehensive care of these patients [12].

## 5. Conclusions

Preterm delivery and abdominal pain are potential predictors for adverse neonatal outcomes in patients with complete uterine rupture. However, in preterm patients with abdominal pain, making a quick decision regarding cesarean delivery is challenging, as the typical signs and symptoms of uterine rupture are difficult to distinguish from those of preterm labor or the side effects of tocolytic agents. In South Korea, where VBAC is rarely performed, patients suspected of uterine rupture mostly have a history of previous myomectomy or cornual resection and are commonly presented to the triage unit at a preterm gestation. Thus, to prevent neonatal complications and improve outcomes, early detection of uterine rupture, decision-making guided by discussions with the patient, and informing them of the relevant associated factors are important.

## Figures and Tables

**Table 1:** Baseline and obstetrical characteristics.

Characteristics	All Cases (*n* = 22)	Scarred Uterus (*n* = 19)	Unscarred Uterus (*n* = 3)	*p*-Value
Maternal age (years) ^a^	34.1 ± 3.4 (34, 30–44)	34.2 ± 3.6 (34, 30–44)	33.3 ± 6.3 (34, 30–44)	0.81
BMI at delivery (kg/m^2^) ^a^	26.0 ± 3.2 (26.3, 18.9–31.2)	26.2 ± 3.0 (26.2, 21.3–31.2)	25.2 ± 5.4 (28.1, 18.9–28.5)	0.848
Primiparity ^b^	18 (81.8%)	16 (84.2%)	2 (66.7%)	0.47
Mode of pregnancy ^b^				0.571
Natural pregnancy	10 (45.5%)	8 (42.1%)	2 (66.7%)	
IUI or IVF/T-ET	12 (54.5%)	11 (57.9%)	1 (33.3%)	
History of uterine surgery ^c^				<0.001
Previous cesarean delivery	2 (9.1%)	2 (10.5%)	0 (0%)	
Previous myomectomy	10 (45.5%)	10 (52.6%)	0 (0%)	
Previous cornual resection	6 (27.3%)	6 (31.6%)	0 (0%)	
Hysteroscopic synechiolysis	1 (4.5%)	1 (5.3%)	0 (0%)	
None	3 (13.6%)	0 (0%)	3 (100%)	
Gestational age at delivery (weeks) ^a^	35.8 ± 3.6 (37.2, 24.4–39.3)	35.3 ± 3.6 (36.6, 24.4–39.1)	39.1 ± 0.2 (39.1, 39.0–39.3)	0.01
Preterm delivery (<37weeks) ^b^	11 (50%)	11 (57.9%)	0 (0%)	0.214
Mode of delivery ^b^				0.01
Vaginal delivery	1 (4.5%)	0 (0%)	1 (33.3%)	
Cesarean delivery	21 (95.5%)	19 (100%)	2 (66.7%)	
Twin pregnancy ^b^	3 (13.6%)	3 (15.8%)	0 (0%)	1
Associated uterine anomaly ^b^	1 (4.5%)			
Symptoms and signs (multiple) ^c^				
None	5 (22.7%)	5 (26.3%)	0 (0%)	1
Abdominal pain	16 (72.7%)	14 (73.7%)	2 (66.7%)	1
Vaginal bleeding	1 (4.5%)	0 (0%)	1 (33.3%)	0.136
Hemodynamic instability	4 (18.2%)	3 (15.8%)	1 (33.3%)	0.47
Fetal heart rate abnormality	5 (22.7%)	5 (26.3%)	0 (0%)	1
Site of uterine rupture ^c^				0.002
Fundus	8 (36.4%)	8 (42.1%)	0 (0%)	
Ant/post wall	7 (31.8%)	7 (36.8%)	0 (0%)	
Cornus	5 (22.7%)	4 (21.1%)	1 (33.3%)	
Lower segment	2 (9.1%)	0 (0%)	2 (66.7%)	
Time interval of symptoms/signs to delivery or detection (min) ^a^	79.6 ± 82.8 (64.5, 0–282)	90.3 ± 84.0 (77, 0–282)	12.0 ± 20.8 (0, 0–36)	0.089

Data presented with mean ± standard deviation (median, range) or number (percent). BMI, body mass index; IUI, intrauterine insemination; IVF, in vitro fertilization; T-ET, thawing-embryo transfer; FD, fetal demise; ^a^ Mann–Whitney U-test, ^b^ Fisher’s exact test, ^c^ Chi-square test.

**Table 2:** Comparison of maternal and neonatal outcomes in scarred and unscarred uterus.

Characteristics	All Cases (*n* = 22)	Scarred Uterus (*n* = 19)	Unscarred Uterus (*n* = 3)	*p*-Value
EBL (mL) ^a^	993.2 ± 657.6 (800, 500–3000)	960.5 ± 658.0 (800, 500–3000)	1200.0 ± 755.0 (1100, 500–2000)	0.561
Blood transfusion ^b^	9 (40.9%)	7 (36.8%)	2 (66.7%)	0.544
Bladder injury ^b^	1 (4.5%)	1 (5.3%)	0 (0%)	1.000
Hysterectomy ^b^	0 (0.0%)	0 (0%)	0 (0%)	-
DIC ^b^	4 (18.2%)	2 (10.5%)	2 (66.7%)	0.073
Maternal death ^b^	0 (0.0%)	0 (0%)	0 (0%)	-
Birth weight (gram)	2545.9 ± 657.9 (2540, 690–3250)	2456.8 ± 664.2 (2500, 690–3240)	3110.0 ± 145.3 (3120, 2960–3250)	0.077
Preterm birth ^b^	11 (50.0%)	11 (57.9%)	0 (0%)	0.214
Apgar score at 1 min (≤7) ^b^	12 (54.5%)	11 (57.9%)	1 (33.3%)	0.571
Apgar score at 5 min (≤7) ^b^	8 (36.4%)	8 (42.1%)	0 (0%)	0.273
NICU admission ^b^	10 (45.5%)	9 (47.4%)	1 (33.3%)	1.000
FD or perinatal death ^b^	4 (18.2%)	4 (21.1%)	0 (0%)	1.000

Data presented with mean ± standard deviation (median, range) or number (percent). EBL, estimated blood loss; DIC, disseminated intravascular coagulation; NICU, neonatal intensive care unit; FD, fetal demise; ^a^ Mann–Whitney U-test, ^b^ Fisher’s exact test.

**Table 3:** Associated factors with adverse neonatal outcomes.

Characteristics	All Cases (*n* = 22)	No Neonatal Complications (*n* = 10)	NICU Admission or Perinatal Death (*n* = 12)	*p*-Value
Maternal age (years) ^a^	34.1 ± 3.4 (34, 30–44)	33.7 ± 3.2 (33.5, 30–38)	34.4 ± 3.7 (34.5, 30–44)	0.791
BMI at delivery (kg/m^2^) ^a^	26.0 ± 3.2 (26.3, 18.9–31.2)	26.3 ± 3.0 (27.4, 21.3–29.7)	25.8 ± 3.5 (25.9, 18.9–31.2)	0.621
Primiparity ^b^	18 (81.8%)	8 (80%)	10 (83.3%)	1
Mode of pregnancy ^b^				0.391
Natural pregnancy	10 (45.5%)	6 (60%)	4 (33.3%)	
IUI or IVF/T-ET	12 (54.5%)	4 (40%)	8 (66.7%)	
History of uterine surgery^c^				0.815
Previous cesarean delivery	2 (9.1%)	1 (10%)	1 (8.3%)	
Previous myomectomy	10 (45.5%)	4 (40%)	6 (50%)	
Previous cornual resection	6 (27.3%)	3 (30%)	3 (25%)	
Hysteroscopic synechiolysis	1 (4.5%)	0 (0%)	1 (8.3%)	
None	3 (13.6%)	2 (20%)	1 (8.3%)	
Scarred uterus ^b^	19	8 (80%)	11 (91.7%)	0.571
Gestational age at delivery (weeks) ^a^	35.8 ± 3.6 (37.2, 24.4–39.3)	38.1 ± 0.9 (38.2, 36.6–39.3)	33.9 ± 3.9 (34.0, 24.4–39.1)	0.006
Preterm delivery (<37weeks) ^b^	11 (50%)	2 (20%)	9 (75%)	0.03
Mode of delivery ^b^				0.455
Vaginal delivery	1 (4.5%)	1 (10%)	0 (0%)	
Cesarean delivery	21 (95.5%)	9 (90%)	12 (100%)	
Twin pregnancy ^b^	3 (13.6%)	0 (0%)	3 (25%)	0.221
Associated uterine anomaly ^b^	1 (4.5%)	1 (10%)	0 (0%)	1
Symptoms and signs (multiple) ^c^				
None	5 (22.7%)	4 (40%)	1 (8.3%)	0.135
Abdominal pain	16 (72.7%)	5 (50%)	11 (91.7%)	0.056
Vaginal bleeding	1 (4.5%)	1 (10%)	0 (0%)	0.455
Hemodynamic instability	4 (18.2%)	1 (10%)	3 (25%)	0.594
Fetal heart rate abnormality	5 (22.7%)	0 (0%)	5 (41.7%)	0.04
Site of uterine rupture ^c^				0.726
Fundus	8 (36.4%)	4 (40%)	4 (33.3%)	
Ant/post wall	7 (31.8%)	2 (20%)	5 (41.7%)	
Cornus	5 (22.7%)	3 (30%)	2 (16.7%)	
Lower segment	2 (9.1%)	1 (10%)	1 (8.3%)	
Time interval of symptoms/signs to delivery or FD detection (min) ^a^	79.6 ± 82.8 (64.5, 0–282)	42.0 ± 63.8 (0, 0–192)	111 ± 85.9 (92.5, 0–282)	0.032
EBL (mL) ^a^	993.2 ± 657.6 (800, 500–3000)	800 ± 454.6 (700, 500–2000)	1154.2 ± 770.9 (800, 600–3000)	0.089

Data presented with mean ± standard deviation (median, range) or number (percent). BMI, body mass index; IUI, intrauterine insemination; IVF, in vitro fertilization; T-ET, thawing-embryo transfer; FD, fetal demise; EBL, estimated blood loss; ^a^ Mann–Whitney U-test, ^b^ Fisher’s exact test, ^c^ Chi-square test.

**Table 4:** Univariable logistic regression analysis of factors associated with adverse neonatal outcomes.

Factors Associated with Adverse Neonatal Outcomes	Univariate	*p*-Value
	OR (95% CI)	
Maternal age (years)	1.068 (0.824–1.385)	0.619
BMI at delivery (kg/m^2^)	0.958 (0.732–1.253)	0.753
Multiparous (vs. primiparous)	0.800 (0.091–7.002)	0.840
IUI or IVF pregnancy (vs. natural pregnancy)	3.000 (0.525–17.159)	0.217
Scarred uterus (vs. nonscarred uterus)	2.750 (0.211–35.838)	0.440
Gestational weeks at delivery	0.429 (0.208–0.883)	0.022
Preterm delivery (vs. full-term delivery)	12.000 (1.581–91.084)	0.016
Twin pregnancy (vs. singleton)	N/A *	0.999
Associated uterine anomaly (vs. No)	N/A *	1.000
Abdominal pain (vs. No)	11.000 (1.005–120.430)	0.050
Vaginal bleeding (vs. No)	N/A *	1.000
Hemodynamic instability (vs. No)	3.000 (0.260–34.575)	0.378
Fetal heart rate abnormality (vs. No)	N/A *	0.999
Time interval of symptoms/signs to delivery or FD detection	0.986 (0.972–1.001)	0.072
EBL (mL)	0.999 (0.997–1.001)	0.246

OR, Odds ratio; CI, confidence interval; BMI, body mass index; IUI, intrauterine insemination; IVF, in vitro fertilization; FD, fetal demise; EBL, estimated blood loss; *: not applicable due to small sample size or zero frequency in one group.

## Data Availability

The data presented in this study are available on request from the corresponding author.
